# The chromosomal genome sequence of a peltospirid gastropod, *Rhynchopelta concentrica* McLean, 1989 (Neomphalida: Peltospiridae) and its associated microbial metagenome sequences

**DOI:** 10.12688/wellcomeopenres.27361.1

**Published:** 2026-08-11

**Authors:** Ian V. Hughes, Stephane Hourdez, Roxanne A. Beinart, Peter Girguis, Graeme Oatley, Elizabeth Sinclair, Eerik Aunin, Noah Gettle, Camilla Santos, Michael Paulini, Haoyu Niu, Victoria McKenna, Rebecca O’Brien, Wellcome Sanger Institute Tree of Life Management, Samples and Laboratory team, Wellcome Sanger Institute Scientific Operations: Sequencing Operations, Wellcome Sanger Institute Tree of Life Core Informatics team, EBI Aquatic Symbiosis Genomics Data Portal Team, Aquatic Symbiosis Genomics Project Leadership

**Affiliations:** 1 Organismic and Evolutionary Biology, Harvard University, Cambridge, Massachusetts, USA; 2 UMR 8222 LECOB, CNRS, Sorbonne University, Observatoire Océanologique de Banyuls,, Banyuls-sur-Mer, France; 3 Graduate School of Oceanography, University of Rhode Island, Narragansett, Rhode Island, USA; 4 Tree of Life Programme, Wellcome Sanger Institute, Hinxton, England, UK

**Keywords:** Rhynchopelta concentrica, peltospirid gastropod, genome sequence, chromosomal, Neomphalida, microbial metagenome assembly

## Abstract

We present a genome assembly from a specimen of *Rhynchopelta concentrica* (peltospirid gastropod; Mollusca; Gastropoda; Neomphalida; Peltospiridae). The genome sequence has a total length of 716.07 megabases. Most of the assembly (92.08%) is scaffolded into 15 chromosomal pseudomolecules. The mitochondrial genome has also been assembled, with a length of 15.75 kilobases. From the metagenome data, we recovered nine bins, of which four were high-quality MAGs.

## Species taxonomy

Eukaryota; Opisthokonta; Metazoa; Eumetazoa; Bilateria; Protostomia; Spiralia; Lophotrochozoa; Mollusca; Gastropoda; Neomphalina; Neomphalida; Neomphaloidea; Peltospiridae; *Rhynchopelta*; *Rhynchopelta concentrica* McLean, 1989 (NCBI:txid55023).

## Background

Peltospiridae is a family of gastropods found at hydrothermal vents around the globe. Peltospirids vary considerably in shell morphology, ranging from limpet-shaped to coiled forms ( Fretter, 1989). *Rhynchopelta concentrica* McLean, 1989 (Gastropoda: Neomphalida: Peltospiridae) is a large (up to 12.6 mm) limpet-shaped peltospirid gastropod, with a known distribution from 21° N to 9.5° N at hydrothermal vents along the East Pacific Rise ( McLean, 1989; Mills *et al.*, 2007). *Rhynchopelta concentrica* can be identified by its large size and fine, concentric ribs along the dorsal surface of the shell ( McLean, 1989). Individuals are often abundant on the chitinous tubes of *Riftia pachyptila* worms ( McLean, 1989), where they are hypothesised to feed primarily on particulate organic material ( Gaudron *et al.*, 2015). Individuals of *R. concentrica* are gonochoristic, probably reproduce through internal fertilisation, and show continuous oocyte production ( Tyler *et al.*, 2008).

Although chemosynthetic symbionts have been documented in several Peltospiridae taxa, including *Chrysomallon squamiferum* ( Chen *et al.*, 2015a) and *Gigantopelta aegis* ( Chen *et al.*, 2015b), bacterial symbionts have not been reported in the majority of peltospirid gastropods, including *R. concentrica.* Previous studies of peltospirid gastropods have suggested that chemosymbiosis evolved independently several times in this lineage, rather than being ancestral and subsequently lost repeatedly ( Chen *et al.*, 2017). Recent work on peltospirid gastropods has focused primarily on several symbiotic lineages. As an apparently aposymbiotic species, *Rhynchopelta concentrica* therefore provides a useful comparison with related, often co-occurring symbiotic gastropods. These data will provide insights into genomic adaptations associated with the evolution of bacterial symbiosis.

*Rhynchopelta concentrica* was assessed for the IUCN Red List of Threatened Species in 2021 and is listed as Near Threatened because of potential habitat loss from proposed seabed mining activities ( Molloy & Thomas, 2021).

We present a chromosome-level genome sequence for *Rhynchopelta concentrica.* The assembly was produced using the Tree of Life pipeline from a specimen collected at Crab Spa Vent on the East Pacific Rise, in international waters of the Pacific Ocean ( Figure 1). This assembly was generated as part of the Aquatic Symbiosis Genomics project.

**Figure 1.**
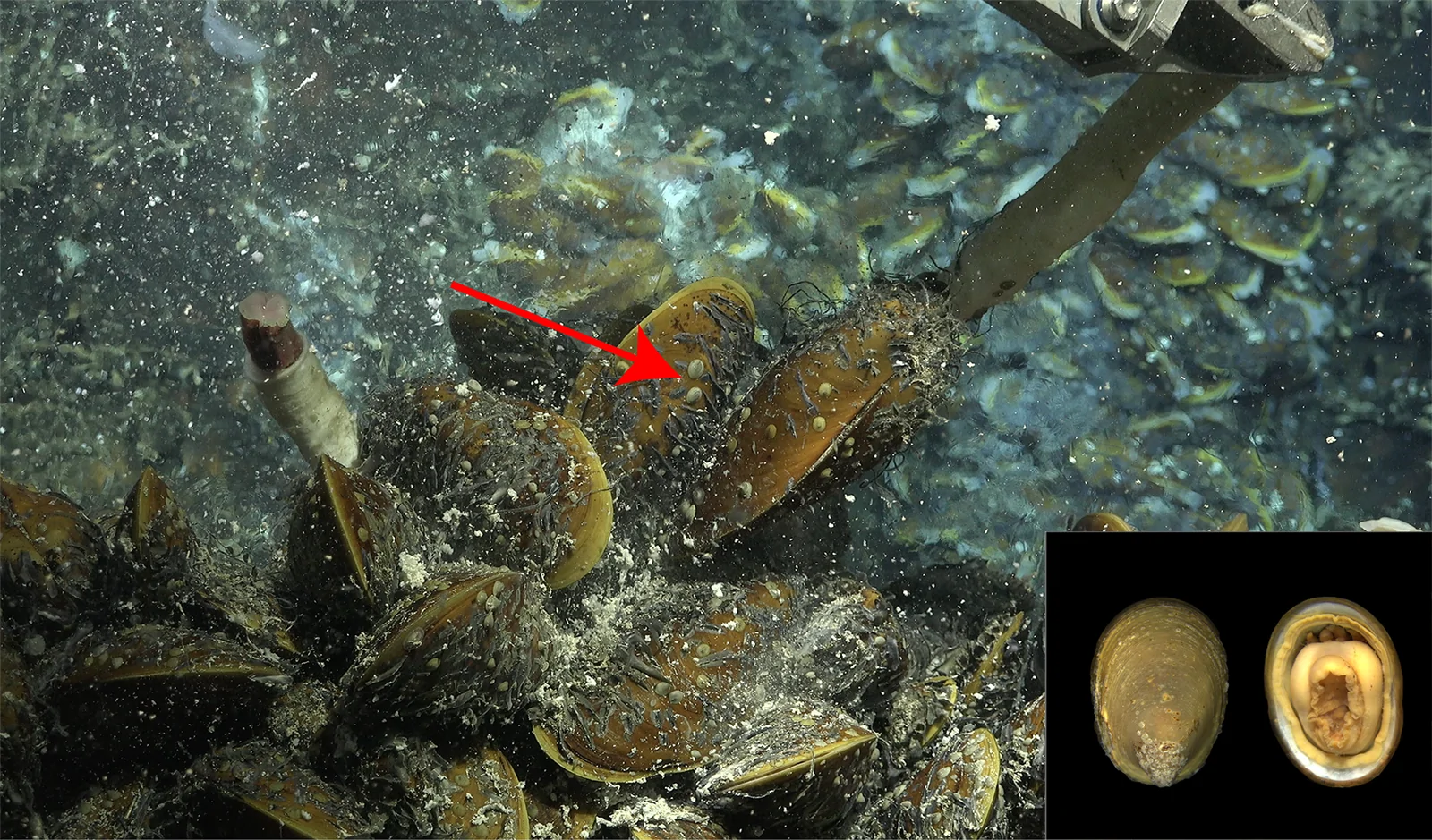
Photograph taken in situ during ROV SuBastian Dive 551. Several species of *Lepetodrilus* limpets are visible in the image; the red arrow indicates a pair of *R. concentrica* limpets. Inset: photograph of a representative *Rhynchopelta concentrica* specimen preserved in 100% ethanol. This specimen was collected alongside the specimen used in this study. The shell of this individual is 8.6 mm long.

## Methods

### Sample acquisition

*Rhynchopelta concentrica* specimens were collected from the bases of *R. pachyptila* tubeworms and from *Bathymodiolus* sp. mussel clumps (latitude 9.8399, longitude −104.2915) on the East Pacific Rise, near Crab Spa Vent. Samples were collected using the remotely operated vehicle (ROV) *SuBastian* during Dive 551 from R/V *Falkor (too)*, during expedition Fkt230629. Specimens were sorted in a 4 °C cold room immediately after recovery, flash-frozen in liquid nitrogen and stored at −80 °C. Individuals were identified by Stephane Hourdez and Ian Hughes.

### Nucleic acid extraction

Protocols for high molecular weight (HMW) DNA extraction developed at the Wellcome Sanger Institute (WSI) Tree of Life Core Laboratory are available on protocols.io ( Howard *et al.*, 2025). The xgRhyConc1 sample was weighed and triaged to determine the appropriate extraction protocol. Tissue from the whole organism was homogenised by cryogenic disruption using the Covaris cryoPREP ^®^ Automated Dry Pulverizer. DNA was extracted by following the Nanobind tissue big DNA protocol. DNA was sheared into an average fragment size of 12–20 kb following the Megaruptor®3 for LI PacBio protocol. Sheared DNA was purified by automated SPRI (solid-phase reversible immobilisation). The concentration of the sheared and purified DNA was assessed using a Nanodrop spectrophotometer and Qubit Fluorometer using the Qubit dsDNA High Sensitivity Assay kit. Fragment size distribution was evaluated by running the sample on the FemtoPulse system. For this sample, the final post-shearing DNA had a Qubit concentration of 5.2 ng/μL and a fragment size of 6.1 kb. The 260/280 spectrophotometric ratio was 1.04, and the 260/230 ratio was 2.2.

RNA was extracted from whole organism tissue of the xgRhyConc3 specimen in the Tree of Life Laboratory at the WSI using the RNA Extraction: Automated MagMax™ *mir*Vana protocol. The RNA concentration was assessed using a Nanodrop spectrophotometer and a Qubit Fluorometer using the Qubit RNA Broad-Range Assay kit. Analysis of the integrity of the RNA was done using the Agilent RNA 6000 Pico Kit and Eukaryotic Total RNA assay.

### PacBio HiFi library preparation and sequencing

Library preparation and sequencing were performed at the WSI Scientific Operations core. Libraries were prepared using the SMRTbell Prep Kit 3.0 (Pacific Biosciences, California, USA), following the manufacturer’s instructions. The kit includes reagents for end repair/A-tailing, adapter ligation, post-ligation SMRTbell bead clean-up, and nuclease treatment. Size selection and clean-up were performed using diluted AMPure PB beads (Pacific Biosciences). DNA concentration was quantified using a Qubit Fluorometer v4.0 (ThermoFisher Scientific) and the Qubit 1X dsDNA HS assay kit. Final library fragment size was assessed with the Agilent Femto Pulse Automated Pulsed Field CE Instrument (Agilent Technologies) using the gDNA 55 kb BAC analysis kit.

The sample was sequenced on a Revio instrument (Pacific Biosciences, California, USA). Prepared libraries with a molarity greater than 2 nM were normalised to 2 nM, and 15 μL was used for making complexes. For libraries with a molarity below 2 nM, the available 10 μL library volume was used without normalisation. Primers were annealed and polymerases were bound to generate circularised complexes, following the manufacturer’s instructions. Complexes were purified using 1.2× SMRTbell beads, diluted to the Revio loading concentration of 200–300 pM, and spiked with a Revio sequencing internal control. The sample was sequenced on a Revio 25 M SMRT cell. SMRT Link software (Pacific Biosciences), a web-based workflow manager, was used to configure and monitor the run and to carry out primary and secondary data analysis.

### Hi-C

***Sample preparation and crosslinking***

The Hi-C sample was prepared from 20–50 mg of frozen whole organism tissue from the xgRhyConc2 sample using the Arima-HiC v2 kit (Arima Genomics). Following the manufacturer’s instructions, tissue was fixed and DNA crosslinked using TC buffer to a final formaldehyde concentration of 2%. The tissue was homogenised using the Diagnocine Power Masher-II. Crosslinked DNA was digested with a restriction enzyme master mix, biotinylated, and ligated. Clean-up was performed with SPRISelect beads before library preparation. DNA concentration was measured with the Qubit Fluorometer (Thermo Fisher Scientific) and Qubit HS Assay Kit. The biotinylation percentage was estimated using the Arima-HiC v2 QC beads.

***Hi-C library preparation and sequencing***

Biotinylated DNA constructs were fragmented using a Covaris E220 sonicator and size selected to 400–600 bp using SPRISelect beads. DNA was enriched with Arima-HiC v2 kit Enrichment beads. End repair, A-tailing, and adapter ligation were carried out with the NEBNext Ultra II DNA Library Prep Kit (New England Biolabs), following a modified protocol where library preparation occurs while DNA remains bound to the Enrichment beads. Library amplification was performed using KAPA HiFi HotStart mix and a custom Unique Dual Index (UDI) barcode set (Integrated DNA Technologies). Depending on sample concentration and biotinylation percentage determined at the crosslinking stage, libraries were amplified with 10–16 PCR cycles. Post-PCR clean-up was performed with SPRISelect beads. Libraries were quantified using the AccuClear Ultra High Sensitivity dsDNA Standards Assay Kit (Biotium) and a FLUOstar Omega plate reader (BMG Labtech).

Prior to sequencing, libraries were normalised to 10 ng/μL. Normalised libraries were quantified again to create equimolar and/or weighted 2.8 nM pools. Pool concentrations were checked using the Agilent 4200 TapeStation (Agilent) with High Sensitivity D500 reagents before sequencing. Sequencing was performed using paired-end 150 bp reads on the Illumina NovaSeq X.

### RNA library preparation and sequencing

Libraries were prepared using the NEBNext® Ultra II Directional RNA Library Prep Kit for Illumina (New England Biolabs), following the manufacturer’s instructions. Poly(A) mRNA was isolated from 100 ng of total RNA using oligo (dT) beads, converted to cDNA, and uniquely indexed; 14 PCR cycles were performed. Libraries were prepared with an intended fragment size of 100–300 bp. Libraries were quantified, normalised, and pooled equimolarly to a final concentration of 2.8 nM before dilution to 150 pM for loading. Sequencing was carried out on the Illumina NovaSeq X, generating paired-end reads.

### Genome assembly

Prior to assembly of the PacBio HiFi reads, a database of *k*-mer counts ( *k* = 31) was generated from the filtered reads using FastK. GenomeScope2 ( Ranallo-Benavidez *et al.*, 2020) was used to analyse the *k*-mer frequency distributions, providing estimates of genome size, heterozygosity, and repeat content.

The HiFi reads were assembled using Hifiasm ( Cheng *et al.*, 2021) with the --primary option. The Hi-C reads ( Rao *et al.*, 2014) were mapped to the primary contigs using bwa-mem2 ( Vasimuddin *et al.*, 2019), and the contigs were scaffolded in YaHS ( Zhou *et al.*, 2023) with the --break option for handling potential misassemblies. The scaffolded assemblies were evaluated using Gfastats ( Formenti *et al.*, 2022), BUSCO ( Manni *et al.*, 2021) and MerquryFK ( Rhie *et al.*, 2020).

The mitochondrial genome was assembled using MitoHiFi ( Uliano-Silva *et al.*, 2023). The MitoHiFi reference was the mitochondrial genome of *Melanodrymia galeronae* (NC_085482.1).

### Assembly curation

The assembly was decontaminated using the Assembly Screen for Cobionts and Contaminants ( ASCC) pipeline. TreeVal was used to generate the flat files and maps for use in curation. Manual curation was conducted primarily in PretextView and HiGlass ( Kerpedjiev *et al.*, 2018). Scaffolds were visually inspected and corrected as described by Howe *et al*. (2021). Manual corrections included 50 breaks, 54 joins, and removal of 12 haplotypic duplications. This reduced the scaffold count by 2.2%. The curation process is described at sanger-tol/curation-resources . PretextSnapshot was used to generate a Hi-C contact map of the final assembly.

### Assembly quality assessment

The Merqury.FK tool ( Rhie *et al.*, 2020) was run in a Singularity container ( Kurtzer *et al.*, 2017) to evaluate *k*-mer completeness and assembly quality for the primary and alternate haplotypes using the *k*-mer database ( *k* = 31) computed prior to genome assembly. The analysis outputs included assembly QV scores and completeness statistics.

The genome was analysed using the BlobToolKit pipeline, a Nextflow implementation of the earlier Snakemake version ( Challis *et al.*, 2020). PacBio reads were aligned to the assembly using minimap2 ( Li, 2018) and processed with SAMtools ( Danecek *et al.*, 2021) to generate coverage tracks. The pipeline runs BUSCO ( Manni *et al.*, 2021) using lineages identified from the NCBI Taxonomy ( Schoch *et al.*, 2020). For the three basal BUSCO lineages, eukaryota, bacteria and archaea, BUSCO genes are aligned to the UniProt Reference Proteomes database ( Bateman *et al.*, 2023) using DIAMOND BLASTp ( Buchfink *et al.*, 2021). The genome assembly is divided into chunks based on the density of BUSCO genes from the closest taxonomic lineage, and each chunk is aligned to the UniProt Reference Proteomes database using DIAMOND BLASTx. Sequences without DIAMOND BLASTx hits are extracted using seqtk and aligned to the NT database using BLASTn ( Altschul *et al.*, 1990). The outputs are consolidated into a BlobDir for visualisation in BlobToolKit. The BlobToolKit pipeline was developed using nf-core tooling ( Ewels *et al.*, 2020) and MultiQC ( Ewels *et al.*, 2016), with containerisation through Docker ( Merkel, 2014) and Singularity ( Kurtzer *et al.*, 2017).

### Metagenome assembly

The metagenome assembly was generated using MetaMDBG ( Benoit *et al.*, 2024). PROKKA ( Seemann, 2014) was used to identify tRNAs and rRNAs in each bin, CheckM ( Parks *et al.*, 2015) (checkM_DB release 2015-01-16) was used to assess bin completeness/contamination, and GTDB-Tk ( Chaumeil *et al.*, 2022) (GTDB release 214) was used to taxonomically classify bins. All bins were assessed for quality and categorised as metagenome-assembled genomes (MAGs) if they met the following criteria: contamination ≤5%, presence of 5S, 16S, and 23S rRNA genes, at least 18 unique tRNAs, and either ≥90% completeness or ≥50% completeness with fully circularised chromosomes ( Bowers *et al.*, 2017). Bins that did not meet these thresholds, or were identified as taxonomic replicates of MAGs, were retained as ‘binned metagenomes’ provided they had ≥50% completeness and ≤10% contamination. A taxonomic tree of the bins was constructed from NCBI classifications using ete3 ( Huerta-Cepas *et al.*, 2016) and visualised with matplotlib.

## Genome sequence report

### Sequence data

PacBio sequencing of the *Rhynchopelta concentrica* specimen generated 23.74 Gb (gigabases) from 3.79 million reads, which were used to assemble the genome. GenomeScope2.0 analysis using a *p* = 2 model gave a *k*-mer-based genome size estimate of 929.99 Mb, with a heterozygosity of 0.88% and repeat content of 73.22% ( Figure 2). These estimates guided expectations for the assembly. Based on the estimated genome size, the sequencing data provided approximately 31× coverage. Hi-C sequencing produced 106.28 Gb from 351.92 million reads, which were used to scaffold the assembly. RNA sequencing data were also generated and are available in public sequence repositories. Table 1 summarises the specimen and sequencing details.

**Figure 2.**
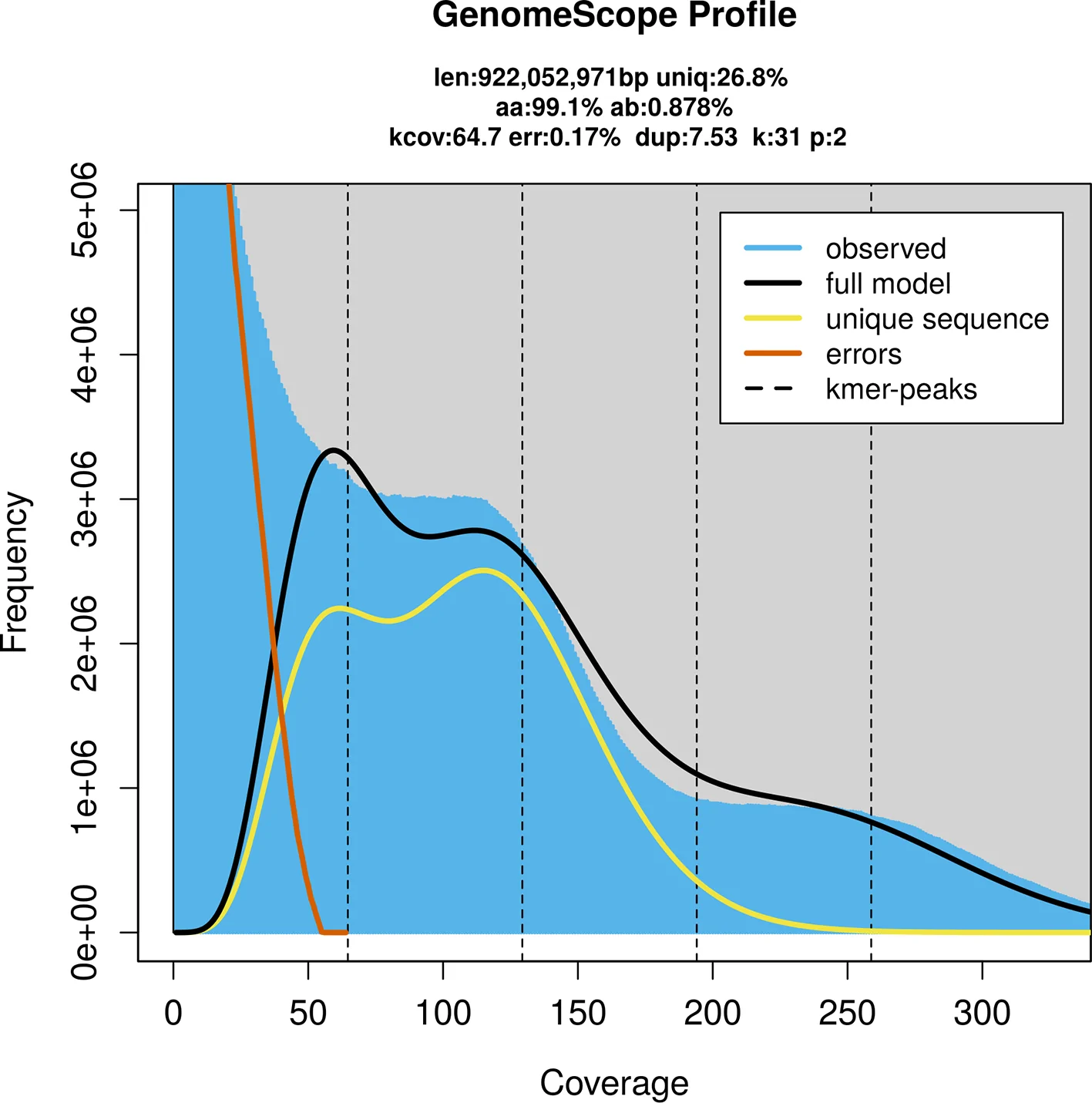
Frequency distribution of *k*-mers generated using GenomeScope2. The plot shows observed and modelled *k*-mer spectra, providing estimates of genome size, heterozygosity, and repeat content based on unassembled sequencing reads.

**Table 1.** Specimen and sequencing data for *Rhynchopelta concentrica* (BioProject PRJEB80241).

Platform	PacBio HiFi	Hi-C	RNA-seq
**ToLID**	xgRhyConc1	xgRhyConc2	xgRhyConc3
**Specimen ID**	URI0000048	URI0000049	URI0000042
**BioSample (source individual)**	SAMEA115701721	SAMEA115701726	SAMEA115701730
**BioSample (tissue)**	SAMEA115701781	SAMEA115701786	SAMEA115701790
**Tissue**	whole organism	whole organism	whole organism
**Instrument**	Revio	Illumina NovaSeq X	Illumina NovaSeq X
**Run accessions**	ERR13698322	ERR13702776	ERR14379139
**Read count total**	3.79 million reads	351.92 million read pairs	42.13 million read pairs
**Base count total**	23.74 Gb	106.28 Gb	12.72 Gb

### Assembly statistics

The primary haplotype was assembled, and contigs corresponding to an alternate haplotype were also deposited in INSDC databases. The final assembly has a total length of 716.07 Mb in 659 scaffolds, with 1 131 gaps, and a scaffold N50 of 57.45 Mb ( Table 2).

**Table 2.** Genome assembly data for *Rhynchopelta concentrica.*

Genome assembly	Primary assembly
**Assembly name**	xgRhyConc1.1
**Assembly accession**	GCA_964300365.1
**Alternate haplotype accession**	GCA_964300395.1
**Assembly level**	chromosome
**Span (Mb)**	716.07
**Number of chromosomes**	15
**Number of contigs**	1 790
**Contig N50**	1.03 Mb
**Number of scaffolds**	659
**Scaffold N50**	57.45 Mb
**Organelles**	Mitochondrial genome: 15.75 kb

Most of the assembly sequence (92.08%) was assigned to 15 chromosomal-level scaffolds. These chromosome-level scaffolds, confirmed by Hi-C data, are named according to size ( Figure 3; Table 3). During genome curation, we observed a region from 65.16 to 67.87 Mbp in Chromosome 3 that is highly repetitive, and the order and orientation of contigs might be uncertain.

**Figure 3.**
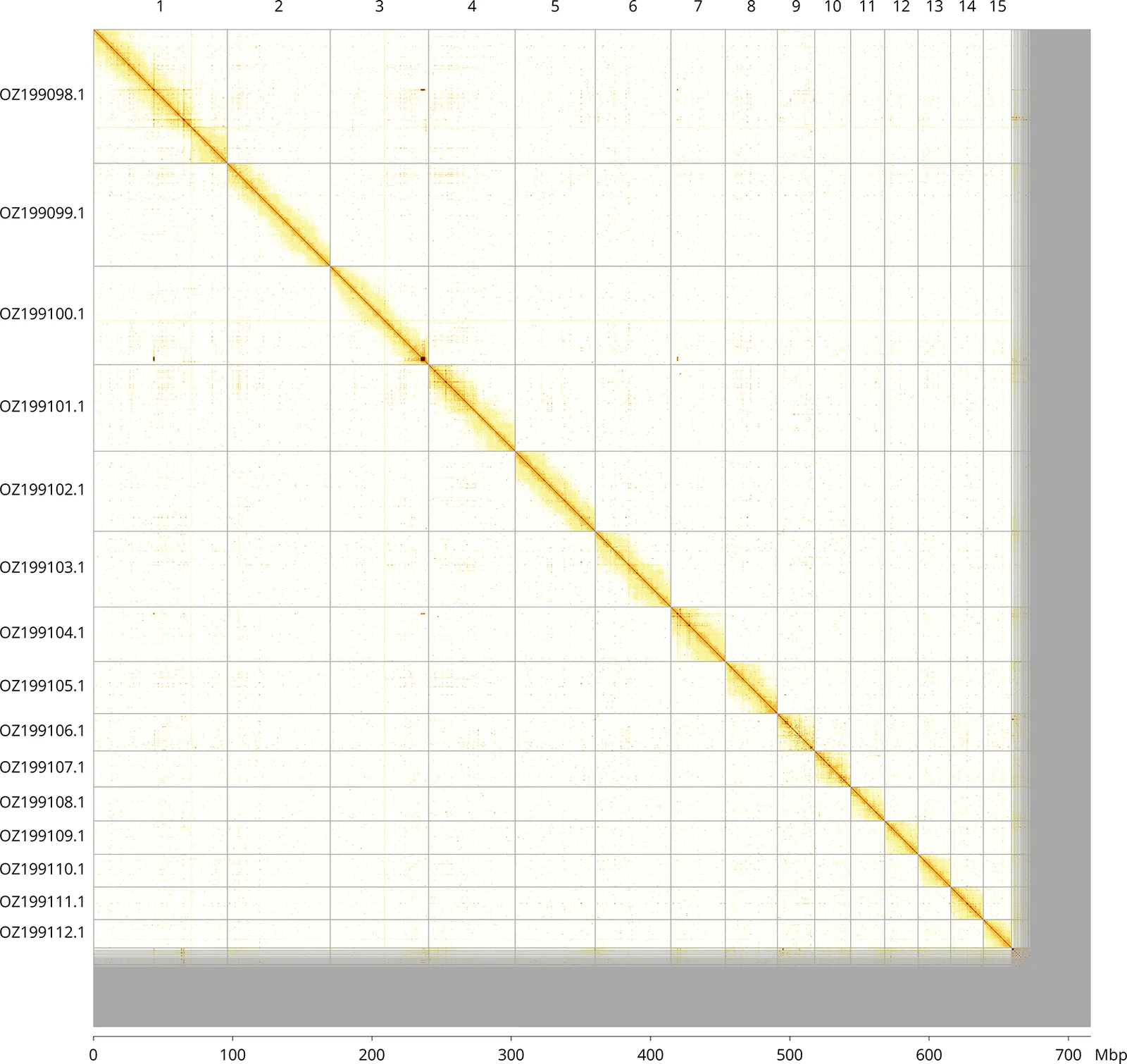
Hi-C contact map of the *Rhynchopelta concentrica* genome assembly. Assembled chromosomes are shown in order of size and labelled along the axes, with a megabase scale shown below. The plot was generated using PretextSnapshot.

**Table 3.** Chromosomal pseudomolecules in the primary genome assembly of *Rhynchopelta concentrica* xgRhyConc1.

INSDC accession	Molecule	Length (Mb)	GC%
OZ199098.1	1	96.23	36.50
OZ199099.1	2	73.80	36.50
OZ199100.1	3	70.82	36.50
OZ199101.1	4	62.02	36.50
OZ199102.1	5	57.45	37
OZ199103.1	6	54.28	37
OZ199104.1	7	39.21	37
OZ199105.1	8	37.36	37
OZ199106.1	9	26.78	38
OZ199107.1	10	25.86	37.50
OZ199108.1	11	24.35	37.50
OZ199109.1	12	23.92	37.50
OZ199110.1	13	23.53	37.50
OZ199111.1	14	23.46	37.50
OZ199112.1	15	20.27	37.50

The mitochondrial genome was also assembled (length 15.75 kb, OZ199113.1). This sequence is included as a contig in the multifasta file of the genome submission and as a standalone record.

### Assembly quality metrics

The combined primary and alternate assemblies achieve an estimated QV of 53.0. The *k*-mer completeness is 76.87% for the primary assembly, 0.23% for the alternate haplotype, and 76.95% for the combined assemblies ( Figure 4).

**Figure 4.**
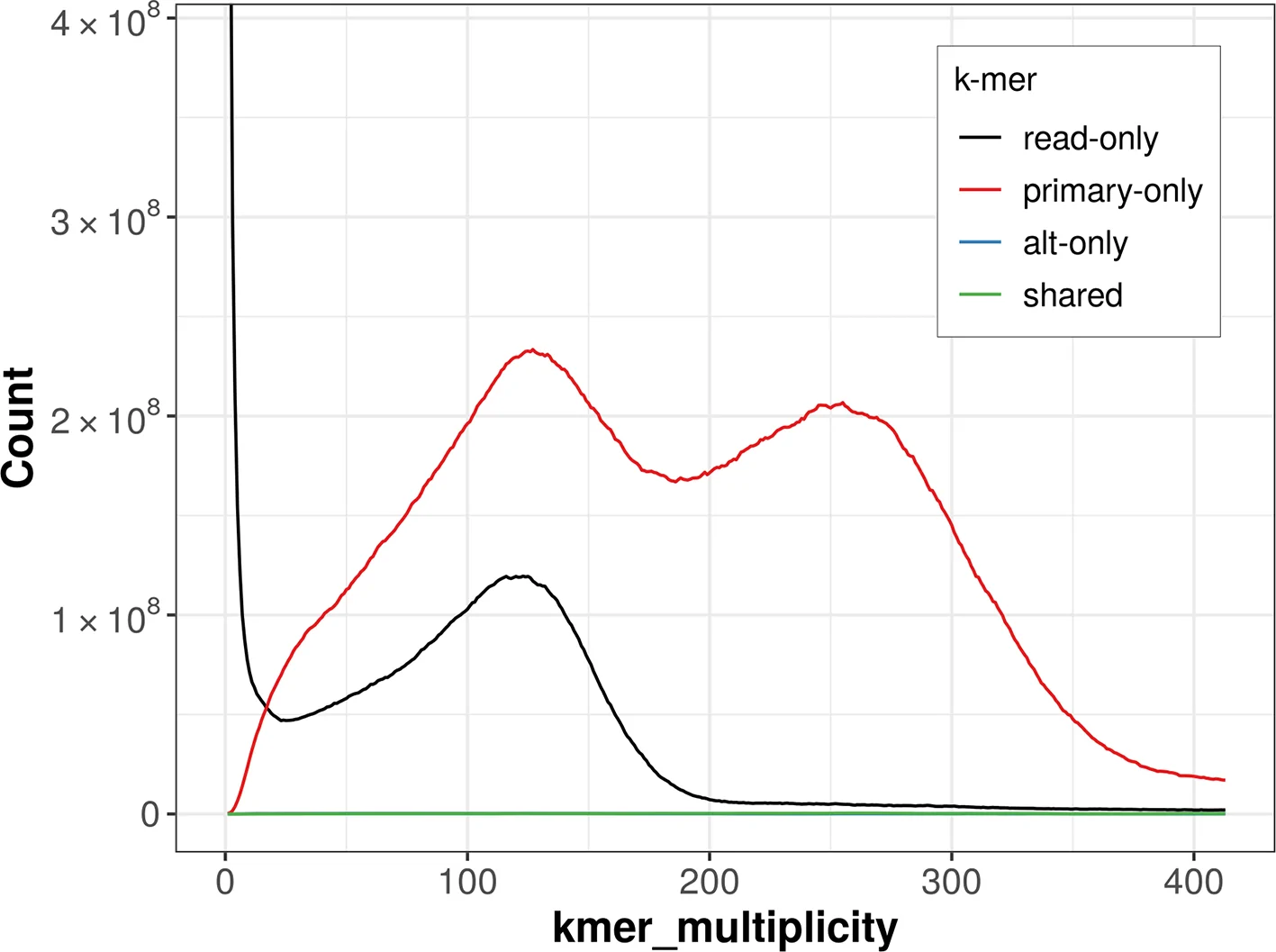
Evaluation of *k*-mer completeness using MerquryFK. This plot illustrates the recovery of *k*-mers from the original read data in the final assemblies. The horizontal axis represents *k*-mer multiplicity, and the vertical axis shows the number of *k*-mers. The black curve represents *k*-mers that appear in the reads but are not assembled. The green curve corresponds to *k*-mers shared by both haplotypes, and the red and blue curves show *k*-mers found only in one of the haplotypes.

BUSCO v.5.5.0 analysis using the mollusca_odb10 reference set ( *n* = 5 295) identified 84.2% of the expected gene set (single = 83.4%, duplicated = 0.7%). The snail plot in Figure 5 summarises the scaffold length distribution and other assembly statistics for the primary assembly. The blob plot in Figure 6 shows the distribution of scaffolds by GC proportion and coverage.

**Figure 5.**
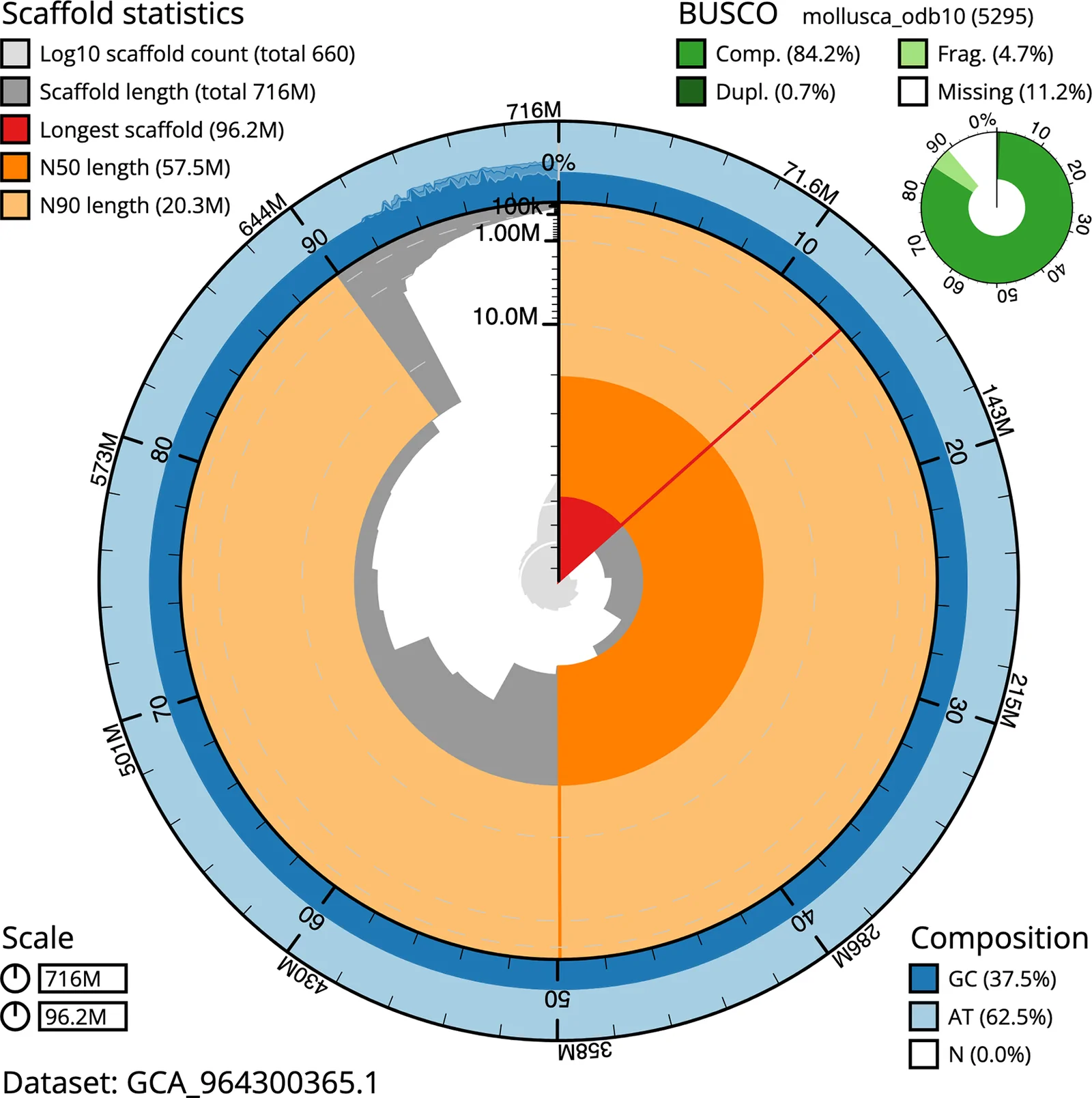
Assembly metrics for xgRhyConc1.1. The BlobToolKit snail plot provides an overview of assembly metrics and BUSCO gene completeness. The circumference represents the length of the whole genome sequence, and the main plot is divided into 1 000 bins around the circumference. The outermost blue tracks display the distribution of GC, AT, and N percentages across the bins. Scaffolds are arranged clockwise from longest to shortest and are depicted in dark grey. The longest scaffold is indicated by the red arc, and the deeper orange and pale orange arcs represent the N50 and N90 lengths. A light grey spiral at the centre shows the cumulative scaffold count on a logarithmic scale. A summary of complete, fragmented, duplicated, and missing BUSCO genes in the mollusca_odb10 set is presented at the top right. An interactive version of this figure can be accessed on the BlobToolKit viewer.

**Figure 6.**
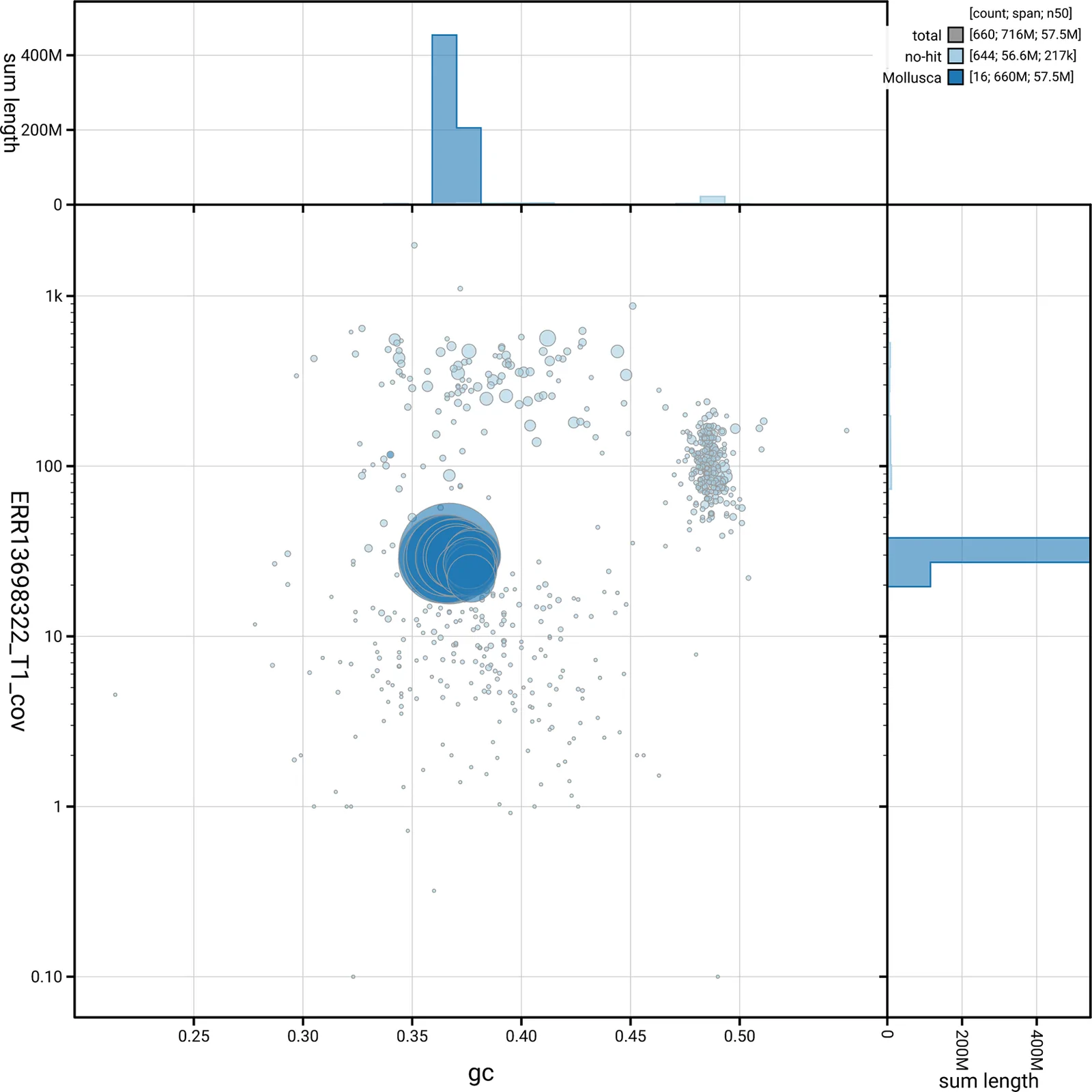
BlobToolKit blob plot for xgRhyConc1.1. The plot shows base coverage (vertical axis) and GC content (horizontal axis). The circles represent scaffolds, with the size proportional to scaffold length and the colour representing phylum membership. The histograms along the axes display the total length of sequences distributed across different levels of coverage and GC content. An interactive version of this figure is available on the BlobToolKit viewer.

Table 4 lists the assembly metric benchmarks adapted from Rhie *et al.* (2021) and the Earth BioGenome Project Report on Assembly Standards January 2026. The EBP metric, calculated for primary assembly, is **6.C.Q53**, meeting the recommended 6.C.Q40 reference standard.

**Table 4.** Earth Biogenome Project summary metrics for the *Rhynchopelta concentrica* assembly.

Measure	Value	Benchmark
EBP summary (primary)	6.C.Q53	6.C.Q40
Contig N50 length	1.03 Mb	≥ 1 Mb
Scaffold N50 length	57.45 Mb	= chromosome N50
Consensus quality (QV)	Primary: 53.0; alternate: 52.5; combined: 53.0	≥ 40
*k*-mer completeness	Primary: 76.87%; alternate: 0.23%; combined: 76.95%	≥ 90%
BUSCO	C:84.2% [S:83.4%, D:0.7%], F:4.7%, M:11.2%, n:5 295	S > 90%; D < 5%
Percentage of assembly assigned to chromosomes	92.08%	≥ 90%

*Note:* The EBP summary uses log10(Contig N50); chromosome-level (C) or log10(Scaffold N50); Q (Merqury QV). BUSCO: C = complete; S = single-copy; D = duplicated; F = fragmented; M = missing; n = orthologues.

### Metagenome report

We recovered nine bins from the metagenome assembly, of which four met the criteria for MAGs. The recovered bins represented four bacterial phyla, with genome sizes ranging from 0.88 to 2.69 Mbp (mean: 1.92 ± 0.52 Mbp). Mean completeness was 87.7% (± 8.6%) with 3.5% (± 3.2%) contamination. Table 5 and Figure 7 summarise the taxa and quality of the metagenome bins.

**Table 5.** Quality metrics and taxonomic assignments of the binned metagenomes.

NCBI taxon	GTDB taxonomy	Quality	Size (bp)	Contigs	Circular	Mean coverage	Compl. (%)	Contam. (%)
*Sulfurovum* sp.	Epsilonproteobacteria	Med.	2085891	89	No	5.20	83.20	7.38
Metamycoplasmataceae bacterium	-	High	879341	1	No	7.96	97.37	0.75
Sulfurovaceae bacterium	Epsilonproteobacteria	Med.	2371697	82	No	4.11	86.61	9.90
*Sulfurovum* sp.	Epsilonproteobacteria	Med.	1728511	60	No	4.36	69.35	4.71
*Sulfurovum* sp.	Epsilonproteobacteria	Med.	2342286	48	No	6.97	80.27	2.87
Sulfurovaceae bacterium	Epsilonproteobacteria	High	2690193	16	No	9.02	97.75	3.42
Gammaproteobacteria bacterium	Gammaproteobacteria	High	1429055	16	No	6.43	91.46	0
Cardiobacteriales bacterium	Gammaproteobacteria	High	2027557	33	No	4.48	90.09	0
Spirochaetia bacterium	Spirochaetia	High	1713607	58	No	3.76	93.20	2.27

**Table 6.** Software versions and sources used for *Rhynchopelta concentrica.*

Software	Version	Source
BLAST	2.14.0	https://ftp.ncbi.nlm.nih.gov/blast/executables/blast+/
BlobToolKit	4.3.9	https://github.com/blobtoolkit/blobtoolkit
BUSCO	5.5.0; 5.7.1	https://gitlab.com/ezlab/busco
bwa-mem2	2.2.1	https://github.com/bwa-mem2/bwa-mem2
CheckM	2015-01-16	https://ecogenomics.github.io/CheckM/
DIAMOND	2.1.8	https://github.com/bbuchfink/diamond
ete3	3.1.3	http://etetoolkit.org
fasta_windows	0.2.4	https://github.com/tolkit/fasta_windows
FastK	1.1	https://github.com/thegenemyers/FASTK
GenomeScope2.0	2.0.1	https://github.com/tbenavi1/genomescope2.0
Gfastats	1.3.6	https://github.com/vgl-hub/gfastats
GTDB-Tk	1.2.1	https://github.com/Ecogenomics/GTDBTk
Hifiasm	0.19.8-r603	https://github.com/chhylp123/hifiasm
HiGlass	1.13.4	https://github.com/higlass/higlass
matplotlib	3.10.3	https://matplotlib.org/
MerquryFK	1.1.0-c1	https://github.com/thegenemyers/MERQURY.FK
MetaMDBG	Pre-release	https://github.com/GaetanBenoitDev/metaMDBG
Minimap2	2.24-r1122	https://github.com/lh3/minimap2
MitoHiFi	3.2.2	https://github.com/marcelauliano/MitoHiFi
MultiQC	1.14; 1.17 and 1.18	https://github.com/MultiQC/MultiQC
Nextflow	23.10.0	https://github.com/nextflow-io/nextflow
PretextSnapshot	0.0.4	https://github.com/sanger-tol/PretextSnapshot
PretextView	1.0.3	https://github.com/sanger-tol/PretextView
Prokka	1.14.5	https://github.com/tseemann/prokka
samtools	1.17; 1.18; 1.19.2; 1.21; 1.6	https://github.com/samtools/samtools
sanger-tol/ascc	0.1.0	https://github.com/sanger-tol/ascc
sanger-tol/blobtoolkit	0.6.0	https://github.com/sanger-tol/blobtoolkit
sanger-tol/curationpretext	1.4.2	https://github.com/sanger-tol/curationpretext
Seqtk	1.4-r122	https://github.com/lh3/seqtk
Singularity	3.9.0	https://github.com/sylabs/singularity
TreeVal	1.1.1	https://github.com/sanger-tol/treeval
YaHS	1.2a.2	https://github.com/c-zhou/yahs

**Figure 7.**
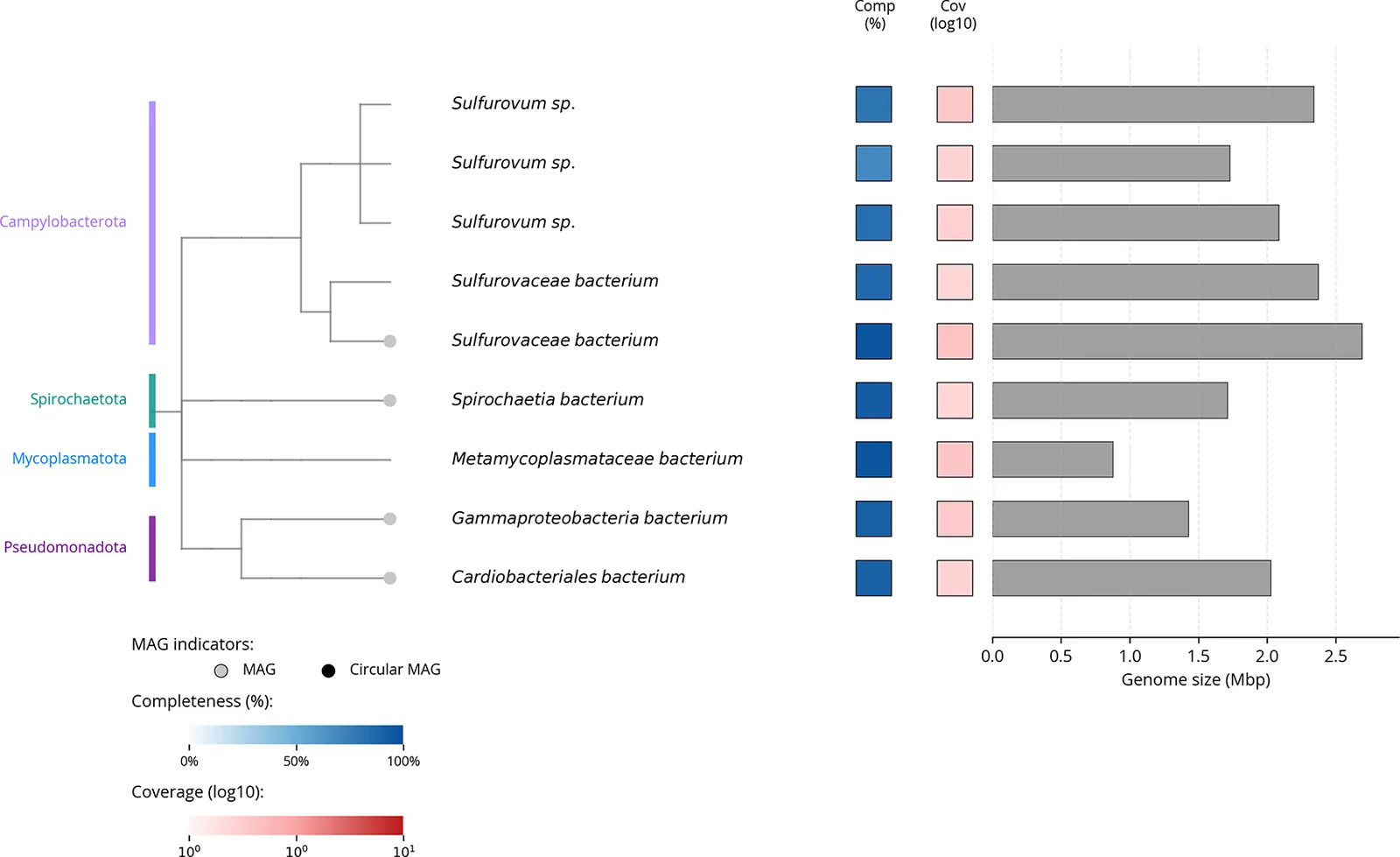
Taxonomic tree based on taxonomic classifications of metagenome bins, constructed using ete3. Colours indicate phylum-level taxonomy. Tracks show genome completeness (blue), sequencing coverage (red, log _10_), and genome size (grey bars, Mbp). High-quality MAGs are marked with grey circles; fully circularised MAGs in black.

## Author information

Contributors are listed at the following links:

• Members of the Wellcome Sanger Institute Tree of Life Management, Samples and Laboratory team
• Members of Wellcome Sanger Institute Scientific Operations – Sequencing Operations
• Members of the Wellcome Sanger Institute Tree of Life Core Informatics team
• Members of the EBI Aquatic Symbiosis Genomics Data Portal Team
• The Aquatic Symbiosis Genomics Project leadership

## Wellcome Sanger Institute – Legal and Governance

The materials that have contributed to this genome note have been supplied by a Tree of Life collaborator. The Wellcome Sanger Institute employs a process whereby due diligence is carried out proportionate to the nature of the materials themselves, and the circumstances under which they have been/are to be collected and provided for use. The purpose of this is to address and mitigate any potential legal and/or ethical implications of receipt and use of the materials as part of the research project, and to ensure that in doing so we align with best practice wherever possible.

The overarching areas of consideration are:

• Ethical review of provenance and sourcing of the material
• Legality of collection, transfer and use (national and international)

Each transfer of samples is undertaken according to a Research Collaboration Agreement or Material Transfer Agreement entered into by the Tree of Life collaborator, Genome Research Limited (operating as the Wellcome Sanger Institute) and in some circumstances other Tree of Life collaborators.

## Data Availability

European Nucleotide Archive: Rhynchopelta concentrica. Accession number PRJEB80241; https://identifiers.org/ena.embl/PRJEB80241. The genome sequence is released openly for reuse. The *Rhynchopelta concentrica* genome sequencing initiative is part of the Aquatic Symbiosis Genomics Project (PRJEB43743) and the Sanger Institute Tree of Life Programme (PRJEB43745). All raw sequence data and the assembly have been deposited in INSDC databases. The genome will be annotated using available RNA-Seq data and presented through the Ensembl pipeline at the European Bioinformatics Institute. Raw data and assembly accession identifiers are reported in Tables 1 and 2. Production code used in genome assembly at the WSI Tree of Life is available at https://github.com/sanger-tol . Table 6 lists software versions used in this study.
