# Supplementary material for: The chromosomal genome sequence of a peltospirid gastropod, Rhynchopelta concentrica McLean, 1989 (Neomphalida: Peltospiridae) and its associated microbial metagenome sequences
Source: Wellcome Open Res. 2026 Aug 11;11:519. [Version 1] doi: 10.12688/wellcomeopenres.27361.1 (PMC13768707; doi:10.12688/wellcomeopenres.27361.1)
Supplement: Peer review history

# Reviewer report: Reviewer response for version 1

**DOI:** 10.21956/wellcomeopenres.30116.r171053

**Published:** 2026-11-13

**Authors:** Masa-aki Yoshida

**Affiliations:** 1 Shimane University, Oki, Japan

The authors present a highly contiguous genome assembly of Rhynchopelta concentrica, a scientifically important species as a hydrothermal vent organism. The obtained genome assembly has reached a generally high standard and will serve as an important genetic resource for conducting genome biology on this organism.

 Clarifying the following three minor points listed below is expected to further enhance the value of this paper.

1. The abstract should clearly state that this organism originates from hydrothermal vents and has a potential symbiotic relationship with bacteria. As it currently stands, the reason for using this organism in the genome analysis, especially why MAG analysis was specifically conducted, cannot be understood from the abstract, and I am concerned that crucial information will not be conveyed to potential readers.
2. Voucher information for the reference materials, such as the shell specimens of the three xgRhyConc1-3 individuals used in the experiment, is not provided. This information is essential for future traceability.
3. Are the genomic data used as input for the MAG analysis the same as those used for the chromosomal genome analysis in this study? It was difficult to determine from the current description whether they originated from different extracted DNA or if it was a single sample. Was all the tissue, including gill tissue that might harbor symbiotic bacteria, used for DNA extraction? If you conducted analyses to check whether contigs likely derived from symbiotic bacteria obtained from the MAG were mixed into the chromosomal genome, it would be necessary to state that clearly.

Are sufficient details of methods and materials provided to allow replication by others?

Partly

Is the rationale for creating the dataset(s) clearly described?

Partly

Are the datasets clearly presented in a useable and accessible format?

Yes

Are the protocols appropriate and is the work technically sound?

Yes

Reviewer Expertise:

Genomics, Evolutionary Biology

I confirm that I have read this submission and believe that I have an appropriate level of expertise to confirm that it is of an acceptable scientific standard, however I have significant reservations, as outlined above.

# Reviewer report: Reviewer response for version 1

**DOI:** 10.21956/wellcomeopenres.30116.r169528

**Published:** 2026-10-14

**Authors:** Qingxiang Guo

**Affiliations:** 1 Northwestern University Feinberg School of Medicine, Chicago, Illinois, USA

The manuscript reports a chromosome-level genome assembly for Rhynchopelta concentrica together with microbial metagenome data. The dataset should be useful for comparative studies of peltospirids and hydrothermal-vent symbioses. Overall, the manuscript is clear, and the data are well presented, but a few points need clarification. In particular, the relatively low k-mer and BUSCO completeness values should be discussed more carefully, and the metagenome workflow needs enough detail to make the binning analysis reproducible. The use of different individuals for PacBio, Hi-C, and RNA-seq should also be stated more clearly. I consider these points important to address before the resource can be fully evaluated, but they appear to be resolvable with clarification and additional methodological detail.

 1. “GenomeScope2.0 analysis... gave a k-mer-based genome size estimate of 929.99 Mb...”

 The final primary assembly is 716.07 Mb, and both the k-mer completeness (76.87%) and BUSCO completeness (84.2%) are below the benchmarks listed in Table 4. I think this difference deserves some discussion. Is it mainly due to unresolved repetitive sequence, limited sequencing depth, or something else? The very low k-mer completeness of the alternate haplotype (0.23%) should also be explained.

 2. “The Hi-C sample was prepared... from the xgRhyConc2 sample...”

 PacBio, Hi-C, and RNA-seq were generated from three different individuals. This is clear from Table 1, but not obvious from the main text. Since Hi-C from xgRhyConc2 was used to scaffold the PacBio assembly from xgRhyConc1, the authors should briefly explain the use of different individuals.

 3. “The metagenome assembly was generated using MetaMDBG...”

 I could not find enough detail on how the metagenome bins were generated. 

 MetaMDBG is described for assembly, but the binning step itself is unclear. Please provide the binning method or software and indicate which sequencing reads were used for the metagenome assembly.

 4. The title, “...its associated microbial metagenome sequences”

 I would be a little cautious with the term “associated microbial metagenome.” DNA was extracted from whole animals collected directly from a hydrothermal-vent environment, so some of these bacteria could come from the gut, body surface, or surrounding environment rather than representing stable host-associated microbes. A short clarification of this limitation would be appropriate.

 5. “We recovered nine bins from the metagenome assembly, of which four met the criteria for MAGs.”

 The Abstract and Results state that four MAGs were recovered, but Table 5 appears to contain five entries labelled “High”. Please clarify how the four MAGs were defined and what the “High” category in Table 5 represents.

 Minors:

 - “Based on the estimated genome size, the sequencing data provided approximately 31× coverage.” Please check this value against the reported sequence yield and genome-size estimate.

 - Table 2 reports 659 scaffolds, but Figure 5 reports 660 in legend?.

 - The expedition identifier is given as Fkt230629 in the Methods and Fkt230623 in the Acknowledgements. Are they different ones?

 - Table 4, “Earth Biogenome Project” should be “Earth BioGenome Project.”

 - MetaMDBG is listed as “Pre-release” in Table 6. A specific version or commit would improve reproducibility.

 - There is a duplicated comma in the second affiliation: “Banyuls,, Banyuls-sur-Mer.”

Are sufficient details of methods and materials provided to allow replication by others?

Partly

Is the rationale for creating the dataset(s) clearly described?

Yes

Are the datasets clearly presented in a useable and accessible format?

Yes

Are the protocols appropriate and is the work technically sound?

Partly

Reviewer Expertise:

Computational biology, genomics, bioinfomatics

I confirm that I have read this submission and believe that I have an appropriate level of expertise to confirm that it is of an acceptable scientific standard, however I have significant reservations, as outlined above.

# Reviewer report: Reviewer response for version 1

**DOI:** 10.21956/wellcomeopenres.30116.r166458

**Published:** 2026-09-21

**Authors:** Alice B. Dennis

**Affiliations:** 1 University of Namur, Namur, Belgium

This is a high-quality genome assembly from a deep-sea gastropod.  The assembly metrics are all robust, but I would just point out that the k-mer plot (Figure 2) contains a large number of singletons (labeled as errors), so the coverage of some parts of the genome is perhaps a bit low. This should be kept in mind for some analyses in the future (e.g. repeats)

 This assembly also has the nice addition of assembling dominant metagenomes in the data. These are now available for future investigation.

Are sufficient details of methods and materials provided to allow replication by others?

Yes

Is the rationale for creating the dataset(s) clearly described?

Yes

Are the datasets clearly presented in a useable and accessible format?

Yes

Are the protocols appropriate and is the work technically sound?

Yes

Reviewer Expertise:

Molluscan genomics, genome annotation, molecular ecology

I confirm that I have read this submission and believe that I have an appropriate level of expertise to confirm that it is of an acceptable scientific standard.
